# Brachial vein transposition: an alternative to hemodialysis arteriovenous graft

**DOI:** 10.1590/1677-5449.190077

**Published:** 2019-11-18

**Authors:** Guilherme de Castro-Santos, Alberto Gualter Salles, Giuliano Silva dos Anjos, Ricardo Jayme Procópio, Túlio Pinho Navarro

**Affiliations:** 1 Universidade Federal de Minais Gerais – UFMG, Faculdade de Medicina, Departamento de Cirurgia, Belo Horizonte, MG, Brasil.; 2 Universidade Federal de Minas Gerais – UFMG, Hospital das Clínicas, Serviço de Cirurgia Vascular, Belo Horizonte, MG, Brasil.

**Keywords:** brachial vein, graft, fistula first, brachial artery, hemodialysis access, arteriovenous fistula

## Abstract

**Background:**

There is currently a worldwide effort to increase the options for autogenous hemodialysis access.

**Objectives:**

To evaluate patency and complications of brachial vein transposition compared to other autogenous hemodialysis accesses.

**Methods:**

A retrospective evaluation of 43 patients and 45 procedures. Patients who did not have adequate superficial veins according to duplex scanning were allocated to brachial vein transposition. The sample was thus divided in two groups, as follows: A: brachial vein transposition n=10 and B: other autogenous accesses n=35.

**Results:**

There were no statistical differences between the two groups in terms of age diabetes, systemic arterial hypertension, dyslipidemias, arteriopathies, neoplasms, kidney disease stage, donor artery diameter, recipient vein diameter, systolic blood pressure in the operated limb, postoperative ischemia, hematoma, or infection. There were no statistical differences in terms of patency on day 7: A 80% vs. B 90% p=0.6, on day 30: A 80% vs. B 86% p=0.6, or on day 60: A 60% vs. B 80% p=0.22. There were statistical differences between the groups for number of previous fistulae A 1.0 ± 0.44 vs. B 0.6 ± 0.3 p = 0.04 and upper limb edema A: 20% x B 0% p = 0.04. A vein with diameter of less than 3 mm was associated with an increased risk of early occlusion (RR = 8 p = 0.0125). During the study period there were no procedures using grafts.

**Conclusions:**

Transposition of brachial vein is an alternative to arteriovenous graft.

## INTRODUCTION

An autogenous arteriovenous fistula using superficial forearm veins is the first choice for hemodialysis access because of its greater patency, lower rate of infection and lower morbidity and mortality.^1^^,^ 2 The National Kidney Foundation Dialysis Outcomes Quality Initiative (NKF-DOQI) recommends that at least 65% of patients should have an autogenous arteriovenous fistula for access.^3^

Chronic kidney disease requiring dialysis is a serious condition with high mortality and its prevalence is growing exponentially in Brazil. Over the last two decades, the number of patients on hemodialysis has tripled in Brazil, reaching 120 thousand in 2016. Annual mortality can reach 20%, primarily associated with cardiovascular events and sepsis. Infections related to central venous catheters and synthetic grafts contribute to the high sepsis rates.^4^ Strategies to increase use of autologous veins to construct arteriovenous fistulas for hemodialysis are increasingly encouraged.

Autogenous accesses are associated with double the 1-year primary patency and nine times greater 2-year patency when compared with prosthetic accesses.^5^ Over recent years, with the advent of endovascular procedures, secondary patency of hemodialysis grafts has increased, but at a cost that is six times greater than autogenous fistula.^6^

In efforts to increase the prevalence of use of autologous fistulae, Koontz and Hellings,^7^ in 1983, and Bazan and Schanzer^8^, in 2006, described use of brachial vein transposition (in the superficial and anterior directions) as hemodialysis vascular access. Other studies demonstrated increased patency and lower rates of complications of this type of access over the short and long terms, compared with arteriovenous grafts.^1^^,^ 9 The objectives of the present study are to evaluate the patency and complications of brachial vein transposition compared with other autogenous accesses using the standard superficial veins and to present this method as an alternative to synthetic prostheses as access for hemodialysis.

## METHODS

The protocol was evaluated and authorized by the institutional Research Ethics Committee and registered on the Plataforma Brasil. Free and informed consent forms were unnecessary because this is a retrospective, observational, case-control study. All data were analyzed taking precautions to maintain patient confidentiality, protecting patients’ data.

**Figure 1:**
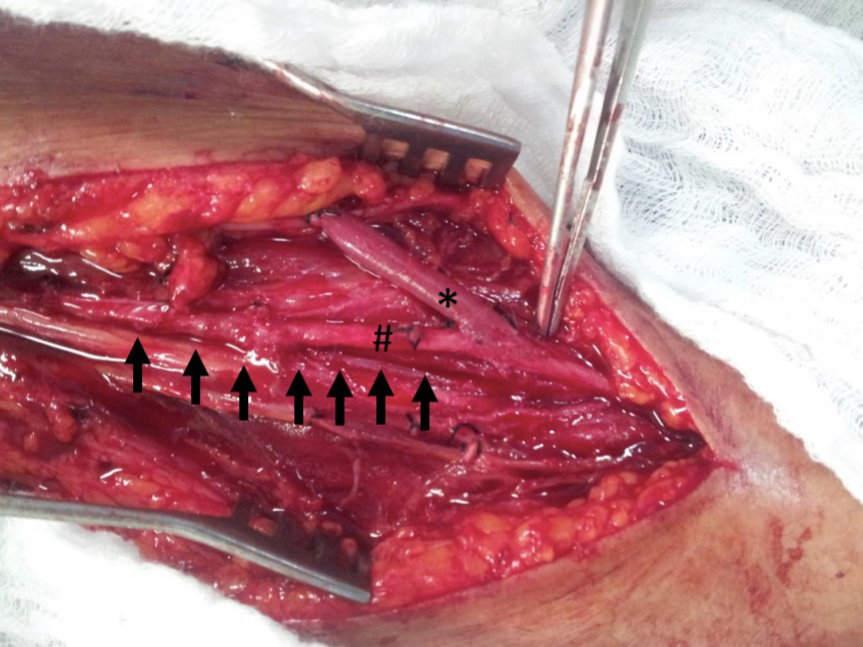
Arteriovenous fistula from the brachial artery to the brachial vein. The brachial vein is indicated with asterisks (*). The brachial artery is indicated with a hash (#). The brachial vein’s anatomic bed is indicated with arrows (the vein itself has been dissected and displaced from its anatomic position).

A retrospective case-control analysis was conducted of patients who had arteriovenous fistulas constructed for hemodialysis from August 2012 to May 2014. These patients were divided into two groups, as follows: Group A: brachial vein transposition (case group); and group B: other types of access (control group). All patients underwent color Doppler ultrasonography examination of arteries and veins for preoperative mapping. In the brachial vein transposition group, surgery was performed using the technique described by Bazan and Schanzer.^8^ After brachial plexus block, an oblique incision was made in the cubital fossa, followed by dissection of the brachial vein and artery. This incision was extended cranially, following the brachial vein longitudinally. The vein was dissected and its tributaries were ligated with 4-0 silk sutures. Shorter tributaries with larger diameters were ligated using 7-0 polypropylene continuous sutures. The brachial vein was then displaced from its bed and a subcutaneous tunnel was opened along the anterior aspect of the arm, into which the vein was transposed (superficial and anterior displacement). An end-to-side anastomosis was constructed between the distal extremity of the vein and the brachial artery in the cubital fossa between the end of the vein and the side of the artery with 7-0 polypropylene, after intra-arterial and intravenous local administration of heparin solution at a proportion of 1:100^8^ (Figure 1).

In the other access group, radiocephalic, brachiocephalic, brachiobasilic, ulnar-basilic and radiobasilic fistulae were constructed according to our routine protocols, with brachial plexus block and with intra-arterial and intravenous local administration of heparin solution at a proportion of 1:100. Brachiobasilic fistulae were constructed during a single intervention with superficial and anterior displacement of the vein.^10^ A range of variables were analyzed, including age, gender, comorbidities, number of previous fistulae, systolic blood pressure in the operated limb, arterial and venous diameters, and kidney disease stage. Patients were followed up at consultations after 7, 30, and 60 days. Postoperative complications such as hematoma, infection, or ischemia were analyzed in both groups. Patency was established by detection of thrill on palpation along the path of the fistula.

Data were expressed as mean (± SD) and counts. Non-categorical variables such as mean age were assessed using Student’s *t* test. The Mann-Whitney U test was used to compare arterial and venous diameters, number of prior surgeries, and systolic pressure in the operated limb. Categorical variables (patency at 7, 30, and 60 days) was studied using the chi-square test with Yates’ correction or Fischer’s test, where appropriate. Results with p < 0.05 were considered statistically significant. All statistical analyses were conducted using Prism 8 for IOS version 8.0.1 (GraphPad Software Inc).

## RESULTS

The sample comprised 43 patients and a total of 45 procedures. Patients were divided into two groups, as follows, Group A: brachial vein transposition, with 10 procedures; and Group B: other types of access, with 35 procedures. In the other accesses group, the following numbers of fistula procedures were conducted: radiocephalic: 16; brachiocephalic: 7; brachiobasilic: 8; ulnar-basilic: 3; and radiobasilic: 1. There were no statistically significant differences between groups in terms of age, diabetes, systemic arterial hypertension, dyslipidemia, arteriopathies, cancer, kidney disease stage, postoperative ischemia, hematoma formation, or infection (Table 1).

**Table 1:** Comparison of individual variables between groups.

	**Group A: brachial vein n = 10**	**Group B: other fistulae n = 35**	**p**
Male sex	6 (60%)	22 (63%)	0.99
Age: minimum, maximum (mean)	8-74 (37.5)	12-78 (42.9)	0.50
Diabetes	4 (40%)	10 (28%)	0.70
Arterial hypertension	6 (60%)	15 (58%)	0.47
Arteriopathies	0	0	-
Dyslipidemia(s)	3 (30%)	10 (28%)	0.99
Neoplasms	0	0	-
Pre-dialytic (kidney disease stage)	7 (70%)	26 (74%)	0.99

**Figure 2:**
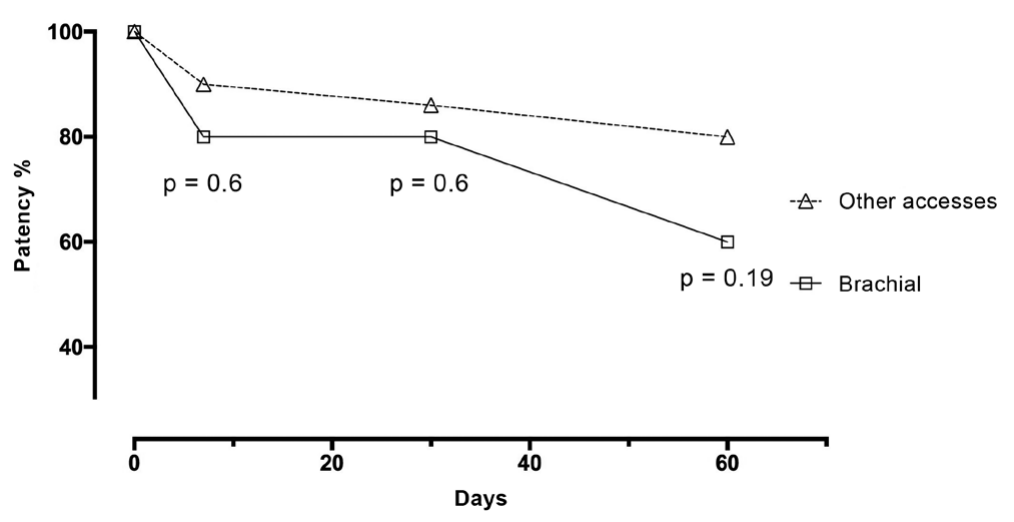
Patency of brachial vein transposition compared to other techniques over time.

There were no differences between the two groups in variables related to anatomy or clinical examination (Table 2). There were no differences in patency at 7 days, A: 80% vs. B: 90%, p = 0.6; 30 days, A: 80% vs. B: 86%, p = 0.6; or 60 days, A: 60% vs. B: 80%, p = 0.22 (Figure 2).

**Table 2:** Comparison of anatomic and clinical examination variables between groups.

	**Group A: brachial vein n = 10**	**Group B: other fistulae n = 35**	**p**
Diameter of donor artery in mm	2.88 ± 0.24	2.83 ± 0.62	0.88
Diameter of recipient vein in mm	3.5 ± 0.77	3.26 ± 0.51	0.49
Systolic blood pressure in the operated limb in mmHg	137 ± 25	142 ± 39	0.76

There was a difference between the groups in terms of number of previous fistulae: A: 1.0 ± 0.44 vs. B: 0.6 ± 0.3, p = 0.04. There was also a difference in upper limb edema at 7 days (A: 20% vs. B: 0%, p = 0.04). The edema was limited to the forearm and had fully resolved by 30 days. Overall patency was 87% at 7 days, 84% at 30 days, and 76% at 60 days. There were no deaths in the brachial vein transposition group. There was one death in the other accesses group (2.86%, p = 0.9 compared with the brachial vein transposition group). Analysis of the patency data for both groups revealed that a donor vein smaller than 3 mm was associated with a 60% 7-day occlusion rate (n = 5). Donor veins exceeding 3 mm had a 7.5% occlusion rate at 7 days (n = 40). A donor vein smaller than 3 mm was associated with an increased risk of early occlusion (RR = 8, p = 0.0125). There was no difference in overall patency between diabetic patients (85.71%) and patients without diabetes (83.87%) at 7 days (n > 0.99).

## DISCUSSION

Over recent years, efforts have been made to reduce use of synthetic prosthetic grafts for definitive hemodialysis access.^11^ Accesses using superficial autogenous veins have lower complication rates and better long-term patency.^4^

Notwithstanding its retrospective nature and the limited number of patients, in this study use of the transposed brachial vein was associated with similar results to other autogenous arteriovenous fistula methods using the customary superficial veins (cephalic and basilic veins). It was observed that 60-day patency was lower with brachial vein transposition when compared with the other autogenous fistulae, although the difference was not statistically significant. This may be related to the low number of patients. Since a trend to lower patency in the brachial vein transposition group was observed, it is possible that statistical significance would have been observed with a larger number of patients. Primary patency at 60 days was 60% with brachial vein transposition, whereas in the other accesses group primary patency was 80%. Several authors have observed similar results for patency. In 2008, Casey et al.^10^ compared brachial vein transposition with transposition of the basilic vein, finding 12-month patency rates of 40% for the brachial vein and 50% for the basilic vein. In 2009, Lioupis et al.^12^ observed 1-year primary patency of 46% in a series of 17 patients. In 2017, Karam et al.^9^ observed 1-year primary patency of 50% in a retrospective study with 64 patients who underwent brachial vein transposition. Patency rates at 2, 3, and 4 years were 42%, 37%, and 27% respectively. In 2017, Pham et al. compared brachial vein transposition with synthetic grafts, observing 1-year primary patency of 62% for brachial vein transposition and 25% for synthetic grafts.^1^ In 2016, Kotsis et al.^13^ conducted a review covering 380 procedures, observing 12-monthy patency rates ranging from 24% to 77%.

Donor vein diameter of less than 3 mm was the greatest predictor of early failure. Several other authors have observed similar results. In 2009, Lauvao et al.^14^ analyzed a range of different factors, finding that vein diameter was the greatest predictor of successful construction of definitive vascular accesses for hemodialysis. A 2016 review by Bashar et al.^15^ also highlighted the importance of using donor veins with adequate caliber and reported a directly proportional relationship between vein caliber and patency.

Patients who underwent brachial vein transposition had undergone a higher number of previous fistula surgeries when compared with those who underwent other surgical methods employing autologous veins. Forty percent of the patients who had brachial vein transposition had already had prior surgery to construct other types of access, compared with 22% in the other accesses group. In 2009, Lioupis et al.^12^ observed that 53% of patients who underwent brachial vein transposition had undergone prior surgery for construction of definitive hemodialysis access. In a 2017 study comparing brachial vein transposition to arteriovenous prostheses, Pham et al.^1^ observed that 28% of the patients who underwent brachial vein transposition had had prior surgery to construct definitive accesses. These findings are to be expected, since in this study, for patients to be allocated to brachial vein transposition, they should not have superficial veins with diameters exceeding 3 mm. Consequently, patients who had already undergone a previous procedure for construction of definitive access were selected for the brachial vein transposition group.

Postoperative edema of the upper limb was observed 7 days after the operation in 20% of the patients who had brachial vein transposition. Patients who underwent other methods of autogenous access construction did not exhibit edema during the same period. Edema had resolved completely by 30 days. This is a very common finding, according to published data. In 2008, Casey et al.^10^ published a retrospective study comparing transposition of the basilic vein to brachial vein transposition, reporting 5.8% edema in the group that underwent brachial vein transposition. In 2005, Angle and Chandra^16^ published a study of 20 patients who underwent brachial vein transposition, observing edema in 5% of them. In 2007, Elwakeel et al.^17^ conducted a study with 21 patients who underwent brachial vein transposition, observing edema in 19%. In a 2009 study with 17 patients, Lioupis et al.^12^ observed edema in 18%. In 2009, Jennings et al.^18^ published a review including 53 patients, reporting postoperative edema in 7%. In 2006, Dorobantu et al.^19^ observed postoperative edema in 34.6% of a series of 33 patients. These findings are to be expected since the brachial vein plays an important role in venous drainage of the arm. However, this edema is not persistent, possibly because of the dense way of venous collaterals in the upper limb.

Transposition of the brachial vein has proven an alternative to using arteriovenous grafts. During the study period, no surgery was performed using prosthetic grafts. Some authors have reported similar results, with reduced use of grafts.^9^^,^ 11 ^,^ 12 ^,^ 17 ^,^ 19 Other authors have compared the results of brachial vein transposition with those of arteriovenous grafts for hemodialysis. In 2017, Pham et al.^1^ compared 29 patients who underwent superficial displacement of the brachial vein and 36 patients who underwent construction of prosthetic arteriovenous access. They observed greater primary patency, at 62%, in the group with brachial vein transposition, compared with 25% in the group with grafts.^1^ However, Torina et al.,^20^ in a 2008 retrospective study with 149 patients observed 25% 1-year primary patency for patients who underwent brachial vein transposition and 50% for patients with access using grafts. In 2009, Lioupis et al.^12^ also compared use of an arteriovenous prosthesis to brachial vein transposition in a retrospective study with 108 patients. Primary patency at 18 months was lower in the brachial vein transposition group, at 27%, compared with 55% for prosthetic arteriovenous access. In both studies, reported brachial vein transposition patency was substantially lower than rates reported by other authors.^1^^,^ 9 ^,^ 11 ^-^ 13

The single intervention surgical technique was chosen, as described by Bazan and Schanzer.^8^ Two-stage surgery for superficial transposition of the brachial vein has been described by several authors. In 2016, Kotsis et al.^13^ published a review in which they observed lower patency among patients who underwent single-stage surgery. One disadvantage of the one-step approach is related to the small diameter of the brachial veins and their structure, which is often delicate and irregular. The fixed anatomy of the brachial vein makes it susceptible to injury during transposition, and this can cause postoperative bleeding, hematoma, stenosis, and thrombosis.^16^ A similar line of reasoning can be applied to superficial transposition of the basilic vein. In 2013, Vrakas et al.^21^ described a 3.2 times greater risk of access failure among patients who underwent single-stage superficial transposition of the basilic vein. The choice of single-stage surgery observed in this study was because of the profile of the patients treated by the public healthcare system. These patients face difficulties that hinder access to health services and a second procedure could have been impossible for some of them.

This study is subject to certain limitations that should be mentioned. It is a retrospective study with a limited follow-up period and a small number of patients. However, the subject is still an ongoing debate in the literature, on which few studies have been published. There is still a knowledge gap in relation to comparisons between brachial vein transposition and use of arteriovenous prostheses. Additional studies are still needed, with larger patient samples and, preferably, prospective and randomized designs.
