# Supplementary material for: Brachial vein transposition: an alternative to hemodialysis arteriovenous graft
Source: J Vasc Bras. 2019 Nov 18;18:e20190077. doi: 10.1590/1677-5449.190077 (PMC6880615; doi:10.1590/1677-5449.190077)
Supplement: Translation

# Translation: Transposição da veia braquial como acesso para hemodiálise: uma alternativa ao uso de prótese sintética

**Authors:** Guilherme de Castro-Santos, Alberto Gualter Salles, Giuliano Silva dos Anjos, Ricardo Jayme Procópio, Túlio Pinho Navarro

**Affiliations:** 1 Universidade Federal de Minais Gerais – UFMG, Faculdade de Medicina, Departamento de Cirurgia, Belo Horizonte, MG, Brasil.; 2 Universidade Federal de Minas Gerais – UFMG, Hospital das Clínicas, Serviço de Cirurgia Vascular, Belo Horizonte, MG, Brasil.

**Keywords:** veia braquial, prótese, fistula first, artéria braquial, aceso para hemodiálise, fístula arteriovenosa

## Abstract

**Contexto:**

Atualmente, observa-se um esforço mundial para aumento do número de acessos autógenos para hemodiálise.

**Objetivos:**

Avaliar a perviedade e as complicações da transposição da veia braquial em comparação aos outros acessos autógenos para hemodiálise.

**Métodos:**

Avaliação retrospectiva de 43 pacientes, com 45 procedimentos. Os pacientes que não apresentaram veias do sistema venoso superficial adequadas ao Duplex Scan pré-operatório foram submetidos à transposição da veia braquial. Esses procedimentos foram divididos em dois grupos: A: uso da veia braquial, n = 10. B: demais acessos, n = 35.

**Resultados:**

Não houve diferença estatística entre os grupos no que se refere à idade, diabetes, hipertensão arterial sistêmica, dislipidemias, arteriopatias, neoplasias, estágio da doença renal, diâmetro da artéria doadora e da veia receptora, pressão arterial sistólica no membro operado, isquemia pós-operatória, formação de hematoma e infecção. Não houve diferença quanto à perviedade aos 7 dias A: 80% vs. B: 90%, p = 0,6; aos 30 dias A: 80% vs. B: 86%, p = 0,6; e aos 60 dias A: 60% vs. B: 80%, p = 0,22. Houve diferença entre os grupos quanto ao número de fístulas prévias A: 1,0 ± 0,44 vs. B: 0,6 ± 0,3, p = 0,04; e quanto ao edema em membro superior A: 20% vs. B: 0%, p = 0,04. A veia doadora menor que 3 mm esteve associada ao maior risco de oclusão precoce (RR = 8, p = 0,0125). Nesse período, não houve nenhum procedimento com o uso de prótese sintética.

**Conclusões:**

A veia braquial transposta é uma alternativa à prótese sintética.

## INTRODUÇÃO

A maior perviedade, a menor taxa de infecção e a menor morbimortalidade fazem da fístula arteriovenosa autógena com veias superficiais em antebraço a primeira escolha entre os acessos para hemodiálise^1^^,^ 2. O National Kidney Foundation Dialysis Outcomes Quality Initiative (NKF-DOQI) recomenda que pelo menos 65% dos pacientes usem as fístulas arteriovenosas autógenas como via de acesso^3^.

A doença renal crônica dialítica é uma condição grave, com alta mortalidade e com crescimento exponencial em nosso meio. Nas últimas duas décadas, observou-se um aumento de três vezes no número de paciente em hemodiálise no Brasil, com 120 mil pacientes em 2016. Observa-se uma mortalidade anual de até 20%, associada principalmente aos eventos cardiovasculares e à sepse. A infecção relacionada ao cateter venoso central e às próteses sintéticas contribui para as altas taxas de sepse^4^. Estratégias visando ao aumento do uso de veias autólogas para a confecção de fístulas arteriovenosas para hemodiálise estão sendo cada vez mais estimuladas.

Observa-se uma perviedade primária duas vezes maior em 1 ano e até nove vezes maior em 2 anos quando se comparam os acessos autógenos às próteses^5^. Nos últimos anos, com o advento dos procedimentos endovasculares, observou-se um aumento da perviedade secundária das próteses para hemodiálise, porém com um custo seis vezes maior quando se compara as fístulas autógenas^6^.

Com o objetivo de aumentar a prevalência do uso das fístulas autólogas, Koontz e Hellings^7^, em 1983, e Bazan e Schanzer^8^, em 2006, descreveram o uso da veia braquial transposta (superficializada e anteriorizada) como acesso vascular para hemodiálise. Outros estudos demonstraram maior perviedade e menores taxas de complicações desse tipo de acesso em curto e longo prazo em comparação às próteses arteriovenosas^1^^,^ 9. Este estudo tem o objetivo de avaliar a perviedade e as complicações da transposição da veia braquial em comparação aos outros acessos autógenos com veias superficiais regulares e apresentá-la como alternativa à prótese sintética como acesso para hemodiálise.

## MÉTODOS

O protocolo foi avaliado e autorizado pelo Comitê de ética em pesquisa da instituição após cadastro na Plataforma Brasil. Não houve necessidade de assinatura de termo de consentimento livre e esclarecido por se tratar de estudo observacional do tipo caso-controle retrospectivo. Todos os dados foram analisados de maneira a manter total sigilo dos dados e da identificação dos pacientes.

Foi realizada uma análise retrospectiva tipo caso-controle dos pacientes submetidos à confecção de fístula arteriovenosa para hemodiálise no período compreendido entre agosto de 2012 e maio de 2014. Esses pacientes foram divididos em dois grupos, a saber: Grupo A: transposição da veia braquial (grupo caso); e grupo B: demais tipos de acessos (grupo controle). Todos os pacientes foram submetidos a eco-Doppler colorido arterial e venoso para mapeamento pré-operatório. No grupo transposição da veia braquial, a cirurgia seguiu a técnica descrita por Bazan e Schanzer^8^. Após o bloqueio de plexo braquial, foi realizada incisão oblíqua em fossa cubital, com dissecção da artéria e da veia braquial. Foi realizado o prolongamento cranial dessa incisão, de maneira longitudinal em topografia da veia braquial, a qual foi dissecada, e suas tributárias foram ligadas com fio seda 4.0. As tributárias mais curtas e de maior diâmetro foram suturadas com pontos contínuos com fio de polipropileno 7.0. A veia braquial foi então mobilizada de seu leito, e foi confeccionado um túnel subcutâneo em face anterior do braço, sobre o qual essa veia foi transposta (superficialização e anteriorização). Foi realizada anastomose entre a extremidade distal da veia e a artéria braquial em fossa cubital de maneira terminal à veia e lateral à artéria com fio de polipropileno 7.0 após administração loco regional intra-arterial e intravenosa de solução de heparina na proporção 1:100^8^ (Figura 1).

**Figura 1:** Fístula arteriovenosa entre a artéria braquial e a veia braquial. A veia braquial está assinalada com asterisco (*). A artéria braquial está assinalada com cerquilha (#). O leito anatômico da veia braquial está assinalado com setas (essa veia foi dissecada e retirada do seu leito anatômico).

No grupo de demais acessos, as fístulas radiocefálica, braquiocefálica, braquiobasílicas, basílico-ulnar e radiobasílicas foram confeccionadas conforme o protocolo do serviço, sob bloqueio do plexo braquial, mediante administração loco regional intra-arterial e intravenosa de solução de heparina na proporção 1:100. As fístulas braquiobasílicas foram confeccionadas em tempo único, com superficialização e anteriorização da veia^10^. Diversas variáveis foram analisadas, como idade, gênero, comorbidades, número de fístulas previamente confeccionadas, pressão arterial sistólica no membro operado, diâmetros arteriais e venosos e estágio da doença renal. Os pacientes foram acompanhados em consultas aos 7, 30 e 60 dias. Complicações pós-operatórias como: hematomas, infecção ou isquemia foram analisadas nos dois grupos. A perviedade foi estabelecida por meio da palpação de frêmito no trajeto da fístula.

Os dados foram apresentados como média (± DP) e números. Variáveis não categóricas foram avaliadas por meio do teste *t* de Student, como as médias de idade. Foi usado o teste U de Mann-Whitney para a comparação do diâmetro arterial e venoso, número de cirurgias prévias e pressão sistólica no membro operado. Variáveis categóricas (perviedade aos 7, 30 e 60 dias) foram estudadas por meio do teste do qui-quadrado com a correção de Yates ou teste de Fischer, quando apropriado. Um valor de p < 0,05 foi considerado estatisticamente significativo. Toda a análise estatística foi realizada por meio do software Prism 8 para IOS versão 8.0.1 (GraphPad Software Inc).

## RESULTADOS

Foram alocados 43 pacientes, com um total de 45 procedimentos. Os pacientes foram divididos em dois grupos, a saber: Grupo A: transposição da veia braquial com 10 procedimentos; e Grupo B: demais tipos de acessos, com 35 procedimentos. No grupo de demais acessos, observou-se o seguinte número de procedimentos: fístula radiocefálica: 16; braquiocefálica: 7; braquiobasílica: 8; basílico-ulnar: 3; e radiobasílica: 1. Não houve diferença estatisticamente significativa entre os grupos quanto à idade, presença de diabetes, hipertensão arterial sistêmica, dislipidemias, arteriopatias, neoplasias, estágio da doença renal, isquemia pós-operatória, formação de hematoma ou infecção (Tabela 1).

**Tabela 1:** Comparação das variáveis individuais nos dois grupos.

	**Grupo A: veia braquial n = 10**	**Grupo B: demais fístulas n = 35**	**Valor de p**
Gênero masculino	6 (60%)	22 (63%)	0,99
Idade: mínimo, máximo (média)	8-74 (37,5)	12-78 (42,9)	0,50
Diabetes	4 (40%)	10 (28%)	0,70
Hipertensão arterial	6 (60%)	15 (58%)	0,47
Arteriopatias	0	0	-
Dislipidemias	3 (30%)	10 (28%)	0,99
Neoplasias	0	0	-
Pré-dialítico (estágio da doença renal)	7 (70%)	26 (74%)	0,99

Não houve diferença em variáveis relacionadas à anatomia ou ao exame clínico em comparação aos dois grupos (Tabela 2). Não houve diferença quanto à perviedade aos 7 dias, A: 80% vs. B: 90%, p = 0,6; aos 30 dias, A: 80% vs. B: 86%, p = 0,6; e aos 60 dias, A: 60% vs. B: 80%, p = 0,22 (Figura 2).

**Tabela 2:** Comparação entre as variáveis anatômicas e de exame clínico nos dois grupos.

	**Grupo A: veia braquial n = 10**	**Grupo B: demais fístulas n = 35**	**Valor de p**
Diâmetro da artéria doadora em mm	2,88 ± 0,24	2,83 ± 0,62	0,88
Diâmetro da veia receptora em mm	3,5 ± 0,77	3,26 ± 0,51	0,49
Pressão arterial sistólica no membro operado em mmHg	137 ± 25	142 ± 39	0,76

**Figura 2:** Perviedade da transposição da veia braquial comparada às outras técnicas em relação ao tempo.

Houve diferença entre os grupos quanto ao número de fístulas prévias, a saber: A: 1,0 ± 0,44 vs. B: 0,6 ± 0,3, p = 0,04. Houve diferença também quanto à presença de edema em membro superior aos 7 dias (A: 20% vs. B: 0%, p = 0,04). Esse edema foi restrito ao antebraço e regrediu completamente aos 30 dias. A perviedade global foi de 87% aos 7 dias, 84% aos 30 dias e 76% aos 60 dias. No grupo de transposição da veia braquial, não houve óbitos. No grupo de demais acessos, houve um óbito (2,86%, p = 0,9) em comparação ao grupo com transposição da veia braquial. Ao analisar os dados de perviedade dos dois grupos em conjunto, observou-se que a veia doadora menor que 3 mm apresentou uma taxa de oclusão aos 7 dias de 60% (n = 5). A veia doadora maior que 3 mm apresentou uma taxa de oclusão de 7,5% aos 7 dias (n = 40). A veia doadora menor que 3 mm esteve associada ao maior risco de oclusão precoce (RR = 8, p = 0,0125). Não houve diferença em termos de perviedade global entre os pacientes diabéticos (85,71%) e não diabéticos (83,87%) aos 7 dias (n > 0,99).

## DISCUSSÃO

Nos últimos anos, observou-se um esforço para a redução do uso de próteses sintéticas como acesso definitivo para hemodiálise^11^. Os acessos com veias autógenas superficiais apresentam menores taxas de complicações e maior perviedade em longo prazo^4^.

Apesar de ser um estudo retrospectivo e com limitado número de pacientes, o uso da veia braquial transposta apresentou resultados semelhantes aos das outras modalidades de fístulas arteriovenosas autógenas com veias superficiais regulares (veias cefálicas e basílicas). Observou-se perviedade menor da transposição da veia braquial aos 60 dias, quando comparada às demais fístulas autógenas, porém sem significado estatístico. Esse resultado pode estar relacionado ao reduzido número de pacientes. Como observou-se uma tendência à menor perviedade do grupo de transposição da veia braquial, poder-se-ia observar significância estatística com um número maior de pacientes. A perviedade primária aos 60 dias foi de 60% com transposição da veia braquial, enquanto no grupo de demais acessos observou-se uma perviedade primária de 80%. Diversos autores encontraram resultados semelhantes quanto à perviedade. Casey et al.^10^, em 2008, ao comparar a transposição da veia braquial à transposição da veia basílica, encontraram perviedade em 12 meses de 40% para a veia braquial e de 50% para a veia basílica. Lioupis et al.^12^, em 2009, observaram uma perviedade primária de 46% em 1 ano em uma série de 17 pacientes. Karam et al.^9^, em 2017, observaram uma perviedade primária de 50% em 1 ano em trabalho retrospectivo, com 64 pacientes submetidos a transposição da veia braquial. A perviedade, nesse estudo, foi de 42%, 37% e 27% aos 2, 3 e 4 anos respectivamente. Pham et al.^1^, em 2017, em trabalho comparando a transposição da veia braquial com prótese sintética, observaram uma perviedade primária de 62% em 1 ano para a transposição da veia braquial e de 25% para a prótese sintética. Kotsis et al.^13^, em 2016, em trabalho de revisão com 380 procedimentos, observaram uma perviedade aos 12 meses entre 24% e 77%.

A veia doadora com um diâmetro menor que 3 mm foi o maior preditor de falência precoce. Diversos outros autores encontraram resultados semelhantes. Lauvao et al.^14^, em 2009, ao analisar diversos fatores, encontraram o diâmetro venoso como maior preditor de sucesso na confecção dos acessos vasculares definitivos para hemodiálise. Uma revisão de Bashar et al.^15^, em 2016, também salientou a importância do uso de veias doadoras com calibres adequados e relacionou uma proporção direta entre o calibre venoso e a perviedade.

Os pacientes submetidos a transposição de veia braquial apresentaram um maior número de cirurgias prévias para fístulas quando comparados aos submetidos a outras modalidades cirúrgicas usando as veias autógenas. Dos pacientes submetidos a transposição da veia braquial, 40% já haviam sido submetidos a cirurgia prévia para confecção de outros acessos em comparação aos 22% no grupo de demais acessos. Lioupis et al.^12^, em 2009, observaram que 53% dos pacientes submetidos a transposição da veia braquial tinham sido submetidos a cirurgias prévias para confecção de acesso definitivo para hemodiálise. Em um trabalho comparando a transposição da veia braquial às próteses arteriovenosas, Pham et al.^1^, em 2017, observaram que 28% dos pacientes submetidos à transposição da veia braquial haviam sido submetidos a cirurgia prévia para acesso definitivo. Esses achados são esperados, uma vez que os pacientes submetidos a transposição da veia braquial neste estudo não deveriam apresentar veias superficiais com diâmetros superiores a 3 mm. Consequentemente, os pacientes que já haviam sido submetidos a algum procedimento para confecção de acessos definitivos prévios foram selecionados para o grupo de transposição da veia braquial.

Observou-se edema pós-operatório em membro superior em 20% dos pacientes submetidos a transposição da veia braquial aos 7 dias do pós-operatório. Os pacientes submetidos às outras modalidades de acessos autógenos não apresentaram edema nesse período. Esse edema regrediu completamente aos 30 dias. Esse achado é bem comum, de acordo com os dados da literatura. Casey et al.^10^, em 2008, em trabalho retrospectivo comparando a transposição da veia basílica à transposição da veia braquial, encontraram 5,8% de edema no grupo submetido a transposição da veia braquial. Angle e Chandra^16^, em 2005, em estudo com 20 pacientes submetidos a transposição da veia braquial, observaram edema em 5% dos pacientes. Elwakeel et al.^17^, em 2007, em estudo com 21 pacientes submetidos a transposição da veia braquial, observaram edema em 19%. Lioupis et al.^12^, em 2009, com 17 pacientes, observaram edema em 18%. Jennings et al.^18^, em 2009, com uma revisão de 53 pacientes, encontraram edema pós-operatório em 7%. Dorobantu et al.^19^, em 2006, observaram 34,6% de edema em pós-operatório em uma série com 33 pacientes. Esses achados são esperados, uma vez que a veia braquial tem um importante papel na drenagem venosa do membro superior. Entretanto, possivelmente devido à densa rede de colaterais venosos em membro superior, esse edema não foi persistente.

A veia braquial transposta foi uma alternativa ao uso da prótese arteriovenosa. Nesse período, não houve nenhuma cirurgia com o uso de prótese. Alguns autores tiveram resultados semelhantes, com redução do uso de próteses^9^^,^ 11 ^,^ 12 ^,^ 17 ^,^ 19. Outros autores compararam os resultados da transposição da veia braquial às próteses arteriovenosas para hemodiálise. Pham et al.^1^, compararam 29 pacientes submetidos à superficialização de veia braquial e 36 pacientes submetidos a confecção de prótese arteriovenosa. Observou-se uma perviedade primária maior, de 62%, no grupo com transposição da veia braquial em comparação a 25% no grupo com prótese^1^. Entretanto, Torina et al.^20^, em 2008, em estudo retrospectivo com 149 pacientes, encontraram uma perviedade primária de 25% em 1 ano para os pacientes submetidos a transposição da veia braquial e de 50% para aqueles submetidos a confecção de prótese. Lioupis et al.^12^, em 2009, também comparou o uso da prótese arteriovenosa à transposição da veia braquial em estudo retrospectivo com 108 pacientes. A perviedade primária em 18 meses foi menor no grupo com transposição da veia braquial, com 27%, em comparação à prótese arteriovenosa de 55%. A perviedade relatada nesses dois trabalhos, principalmente para a transposição da veia braquial, é substancialmente menor quando comparada a outros autores^1^^,^ 9 ^,^ 11 ^-^ 13.

Optou-se pela cirurgia em tempo único, conforme descrito por Bazan e Schanzer^8^. A cirurgia de superficialização da veia braquial foi descrita em dois tempos por diversos autores. Kotsis et al.^13^, em 2016, em revisão sobre o assunto, observaram uma perviedade menor nos pacientes submetidos à cirurgia em estágio único. Uma desvantagem do procedimento de estágio único seria o menor diâmetro das veias braquiais, juntamente com sua estrutura, frequentemente delicada e irregular. A anatomia fixa da veia braquial a torna suscetível a lesões durante a transposição, e isso pode resultar em sangramento pós-operatório, hematoma, estenose e trombose^16^. Raciocínio semelhante pode ser feito para as superficializações da veia basílica. Vrakas et al.^21^, em 2013, descreveram um risco de falência 3,2 vezes maior nos pacientes submetidos à superficialização de veia basílica em tempo único. A escolha de cirurgia em tempo único neste trabalho deveu-se ao perfil dos pacientes tratados pelo sistema público de saúde. Esses pacientes apresentam dificuldade de acesso aos serviços de saúde, e um segundo procedimento poderia ser inviável para alguns deles.

Este estudo apresenta algumas limitações que devem ser citadas. Trata-se de um estudo retrospectivo, com limitado tempo de acompanhamento e reduzido número de pacientes. Entretanto, é um tema ainda em aberto na literatura, com poucos trabalhos publicados. Ainda há uma lacuna no que diz respeito à comparação da veia braquial transposta e ao uso de prótese arteriovenosa. Há ainda a necessidade de mais estudos, com maior número de pacientes e, de preferência, prospectivos e aleatórios.
